# Facile Fabrication of Wood Fiber–Hydrogel Composites for Enhanced Water and Nutrient Efficiency in Soilless Cultivation

**DOI:** 10.3390/ma18235461

**Published:** 2025-12-04

**Authors:** Zhengyong Yang, Yao Qu, Longqing Chen, Huishu Mo, Chunyu Ji, Nicolas Brosse, Mahdi Mubarok, Xiaojian Zhou, Yining Di, Jingjing Liao

**Affiliations:** 1 Key Laboratory of Vegetable Biology of Yunnan Province, College of Landscape and Horticulture, Yunnan Agricultural University, Kunming 650201, China; 2 College of Materials and Chemical Engineering, Southwest Forestry University, Kunming 650224, China; 3 College of Resources and Environment, Yunnan Agricultural University, Kunming 650201, China; 4 Laboratoire d’Etudes et de Recherche sur le Matériau Bois (LERMAB), Faculté des Sciences et Technologies, l’Université de Lorraine, 54000 Vandœuvre-lès-Nancy, France; 5 Faculty of Forestry and Environment, Department of Forest Products, IPB University, Bogor 16680, Indonesia

**Keywords:** hydrogel, soilless substrate, water-use efficiency, nutrient retention, peat replacement

## Abstract

Restrictive regulations on the use of peat and increasing consumption in modern horticulture production have created an irreconcilable contradiction. Wood fibers (WF) produced from forestry residues are considered as a promising peat substitution. However, their poor water- and nutrient-holding capacity limit their application. Here, wood fiber–hydrogel composite (WF-Gel) was developed via a one-pot strategy by grafting poly(acrylic acid-co-acrylamide) (P(AA-co-AM)) onto WF. The structure of the hydrogel network incorporated with WF was confirmed by FTIR spectrophotometry, scanning electron microscopy, X-ray diffractometry, and thermogravimetric analysis. The growing substrate amended with WF-Gel showed higher physical properties, including water-filled porosity (~62.33%) and water-holding capacity (~44.93%) compared with peat incorporated with WF. The pot experiment revealed that WF-Gel significantly increases the chlorophyll content and relative growth rate of choy sum (*Brassica rapa* var. *parachinensis*), especially at the initial transplanting stage. Moreover, choy sum grown in a substrate containing WF-Gel showed a significant increase in biomass accumulation. Additionally, nutrient content and irrigation water-use efficiency data indicated that WF-Gel as a growing medium strongly promotes the water and nutrient efficiency of choy sum. Therefore, the incorporation of this hydrogel modification strategy is a promising approach to promote the water- and nutrient-use efficiency of WF as a soilless substrate component.

## 1. Introduction

The shrinking availability of arable land, along with urbanization, water scarcity, and climate change, is severely impacting agricultural productivity [1]. The use of soilless culture systems for horticulture production has been considered as one of the most promising methods to achieve increased production without damaging the supporting ecosystem [2]. Every year, approximately 40 million m^3^ of peat is consumed as a raw material for horticultural substrate, which supports root growth by providing a sanitary root environment free of plant pathogens and by ensuring good physicochemical properties that allow adequate aeration, water, and nutrient supply [3,4]. However, as peat is partly non-renewable, and peatlands are important carbon storages sites, the use of peat as a growing medium contributes to CO_2_ emissions. Increasing restrictions on the application of peat have accelerated research on innovative, sustainable soilless substrates for horticultural crop production.

In the past decades, many renewable substrate alternatives have been widely investigated as soilless substrate materials in the horticultural industry, such as composts [5], biochars [6], and wood components [7,8]. Among them, such wood substrates can be manufactured from local forest by-products or wood resources using highly efficient engineering techniques. Wood-based substrates are versatile, renewable, and abundantly available [9]. As one of the most promising and innovative wood soilless substrate materials, WF is produced from the defibration of forestry by-products using extruders or disk refiners, and it exhibits excellent air capacity, low bulk density, rewettability, and good draining properties [3,10]. Today, most commercial standard growing media contain a certain proportion of WF. Moreover, the combination of WF with other materials (bark, coir, etc.) can completely replace peat in some cases [1,3,11,12]. However, the inherent limitation of WF in water retention capacity and nitrogen immobilization generally requires a more rigorous fertigation and nutrient management strategy [13,14,15,16,17]. Therefore, challenges remain for using WF at higher proportions or as a stand-alone substrate.

Hydrogel is a three-dimensional network formed by the physical or chemical crosslinking of hydrophilic polymer chains. The excellent water- and nutrient-retention and slow-release characteristics of hydrogel have been investigated for their use as soilless substrates or soil conditioners, notably in agriculture to enhance soil water retention, improve plant water availability, and minimize nutrient leaching [18]. Some researchers have combined such a hydrogel structure with other materials for developing composites with high water- and nutrient-retention capacity. For example, cellulose-based hydrogel composites can be fabricated via grafting polymerization of acrylic acid (AA) onto cellulose to control nutrient release from soil. Similarly, coconut fibers can be functionalized with AA via a graft copolymerization technique, imparting sustained-release properties for nitrogen (N), phosphorus (P), and potassium (K) [19]. Biochar–hydrogel composites have also been prepared, bearing specific features to promote the water- and nutrient-holding capacity [20,21]. This represents a promising pathway to enhance the water- and nutrient-retention capacity of WF as a soilless substrate material. However, such modification techniques have rarely been applied to WF substrates, and their performance in plant growth under soilless cultivation has seldom been evaluated. Moreover, most reported hydrogel–fiber fabrication methods involve multistep pretreatment and sequential polymerization, highlighting the need for simpler and more efficient modification strategies tailored explicitly for WF-based substrates.

In this work, AA and acrylamide (AM) were selected as grafting monomers to modify WF in the presence of the crosslinker MBA, with ammonium persulfate (APS) and potassium persulfate (KPS) as initiators. To simplify the reaction process, a hydrogel prepolymer was first synthesized, directly incorporated with WF, and then cured in an oven to yield the WF-based hydrogel composite (WF-Gel). The optimal copolymerization conditions were investigated through water and saline absorbency, and the results are given in the Supplementary Materials (Figure S1). The main objective of this study was to evaluate the performance of wood fiber (WF) and wood fiber–hydrogel composite (WF-Gel) when used as amendments in peat-based soilless substrates. Pot experiments were conducted by growing leafy vegetables to compare peat alone, peat with 5% WF, and peat with 5% WF-Gel, focusing on their effects on water retention, nutrient availability, and plant growth.

## 2. Materials and Methods

### 2.1. Materials

AA, AM, and MBA were purchased from Macklin Biochemical Technology Co., Ltd. (Shanghai, China). APS and sodium hydroxide (NaOH) were obtained from Kelong Chemical Co., Ltd. (Chengdu, China). KPS and sodium chloride (SC) were supplied by Wokai Biotechnology Co., Ltd. (Shanghai, China) and Sinopharm Chemical Reagent Co., Ltd. (Shanghai, China). WF (60-mesh) was kindly provided by Pu ‘er Futong Fiberboard Co., Ltd. (Pu’er, China). Peat (Pt) was purchased from the local market via Pindstrup and had an electrical conductivity (EC) of 0.757 mS/m, a pH of 5–6, and a particle size range of 1–10 mm. The commercial water-soluble fertilizer (20-20-20, N-P-K, containing micronutrients Fe, Mn, Zn, Cu, and B at 0.2–3%) was purchased from Keze Agricultural Technology Co., Ltd. (Guangdong, China). The above chemicals were all analytical grade and used without purification.

### 2.2. The Preparation of Wood Fiber–Hydrogel Composite (WF-Gel)

P (AA-co-AM) superabsorbent hydrogels have been extensively studied for their potential to enhance plant growth by improving water- and nutrient-use efficiency [22]. Building on this concept, grafting these monomers onto WF followed by chemical crosslinking provides a promising strategy to improve the water and nutrient retention capabilities of the fiber. In the present work, the synthesis followed the procedure reported by [22], with optimal conditions determined from preliminary orthogonal experiments presented in the Supplementary Materials (Figure S1 and Tables S1–S3).

Briefly, 15 g of thermally milled, separated WF was previously dried and mechanically blended with 350 mL of water using an electric whisk at room temperature. Then, 12 g of AM and 31.11 g of neutralized AA (70% neutralization using 25% NaOH) were added to the WF suspension. Then, 0.2 g of initiator mixture APS:KPS (weight ratio of 1:1) and 3 g of crosslinker MBA were slowly added and stirred thoroughly. The reaction was carried out at 80 °C for 1 h under a water bath. After the reaction, the pre-reacted hydrogel polymer was transferred into an oven at 80 °C overnight. Finally, the resulting product was ground into small particles and dispersed in distilled water at room temperature for one day to remove unreacted reagents. The ground hydrogel was separated by filtration and washed repeatedly with distilled water as well as methanol, and finally, dried at 80 °C for one day. The modified WF–hydrogel composite (WF-Gel) was stored in a desiccator for further use.

### 2.3. Plant Materials and Growth Conditions

Choy sum, a commonly consumed Asian leafy vegetable, was used in this work. To obtain a standardized particle size, the WF-Gel was crushed to a diameter of 15–30 mesh using a mill. In this study, the cultivation was divided into two stages. The seeds were initially sown in 50-cavity germination trays containing peat growing substrate, and each tray was irrigated with water prior to sowing. All seedlings were grown in each cavity for 17 days before transplantation. Then, the 17-day-old seedlings were transplanted to 9 cm plastic pots bearing air-dried peat growing substrate, or with the addition of 5 wt% WF or WF–hydrogel composite (WF-Gel), noted as Pt, Pt+5WF, and Pt+5WF-Gel, respectively. The present work will focus on the second stage experiments. All trays were planted under greenhouse conditions located at Yunnan Agriculture University (102°741′ E, 25°12′ N). The experiments were carried out from 14 October to 29 December 2023.

### 2.4. Experimental Design and Treatments

To explore the effects of WF with or without a modification strategy incorporated with peat on plant growth, the same irrigating regimes were performed at the second stage of cultivation from D17 to D38. All transplanted seedlings were subjected to water- and fertilizer-controlled conditions and a top-down irrigation with 60 mL of soluble fertilizer every 10 days.

### 2.5. Characterization of WF and WF-Gel

The chemical structure of WF and WF-Gel was characterized by an FT-IR (Fourier-transform infrared spectroscopy) (Thermo Nicolet IS5, Perkin Elmer, Waltham, MA, USA) in the range of 400–4000 cm^−1^ with a 4 cm^−1^ resolution, using an attenuated total reflection (ATR) model with an average of 32 scans. The surface morphology of WF and WF-Gel was coated with gold and observed using a scanning electron microscope (Zeiss Sigma 300, Oberkochen, Germany). Thermal stability of the samples was determined with a thermo-gravimetric analyzer (TG209F1, NETZSCH, Selb, Germany) in the temperature range from 25 to 600 °C at a heating rate of 20 °C/min under the protection of nitrogen (flow rate = 50 mL/min). All samples were dried before the test. X-ray diffraction (XRD) spectroscopy was performed using a Rigaku Smartlab SE (Tokyo, Japan) in the range of 5–90° with a scanning rate of 5°/min.

### 2.6. Physical and Chemical Properties of WF and WF-Gel Mixing with Peat as the Substrate

Dry bulk density (BD), total porosity (TP), air-filled porosity (AFP), water-filled porosity (WFP) and water-holding capacity (WHC) were analyzed for growing media with methods described in [23,24]. Air-dried substrates were blended with distilled water at a weight ratio of 1:10 and stirred for 6 h at room temperature. The pH and EC of each substrate were determined after filtration using a pH meter (Leici PHB-5, Shanghai, China) and an EC meter (Leici DDB-303A, Shanghai, China), respectively.

### 2.7. Water Retention Capacity

Peat blended with 5 wt% WF or 5 wt% WF-Gel (dried sample = 100 g), as well as the peat control, was immersed in distilled water at room temperature for 24 h. Each substrate blend was filtered and placed in a tinfoil container (diameter = 11 cm; height = 3.8 cm), and the initial weight was recorded as m_1_. The samples were then dried in an oven at 40 °C, and each substrate was weighed every hour and recorded as m_2_. The water retention of each substrate was calculated according to Equation (1).

(1) $$W_{r}=\frac{m_{1}-m_{2}}{m_{1}}\times 100\%$$

### 2.8. Growth Assessment of Plants

The growth assessment of plants in substrates containing WF or WF-Gel was evaluated by biomass analysis at the end of the experiment. The shoot fresh weight of the individual plants was recorded at harvest. Relative growth rate (RGR) was determined using the following Equation (2) [22]:

(2) $$RGR=\frac{ln\;ln\;W_{i}\;-ln\;ln\;W_{f}\;}{t_{f\;-t_{i}}}$$

where W_i_ is the initial shoot fresh weight at D17; W_f_ is the final shoot fresh weight at D38; and t_f_ − t_i_ represents the growth period between the harvesting (D38) and transplanting stage (D17).

### 2.9. Plant Growth Measurement

The growth indicators for choy sum in various substrates were determined by measuring plant height, stem diameter, and number of leaves at D10, D20, and D38. At the same time, biomass data, including aboveground fresh/dry weight and underground fresh/dry weight, were collected at the harvest stage (D38). A SPAD (Soil and Plant Analyzer Development) meter was used to measure chlorophyll content on the second leaf from the top of each choy sum at D10, D20, and D38 (transplanting days 10, 20, and 38).

### 2.10. Nutrient Characterization of Plants

The nutrient analyses of plants were evaluated by using a nutrient analysis photometer (HM-GT4, Hengmei Technology Co., LTD, Weifang City, China) according to the described method procedures. After harvesting (D38), choy sum was dried and milled for further analysis. Five repetitions were conducted in this characterization. Results were then expressed as total amounts of N, P, and K present in the leachate.

### 2.11. Water-Use Efficiency

The irrigation water-use efficiency (Irr WUE) of plants in substrates containing WF or WF-Gel was calculated based on the method of Tan [22]. The aboveground fresh weight at harvest time and the total volume of irrigation water for each plant were recorded. Irrigation water-use efficiency was calculated using Equation (3):

(3) $$IrrWUE=\frac{W_{f}}{V_{t\;}}$$

where W_f_ is the final shoot fresh weight at D38; and V_t_ is the volume of irrigation water from the transplanting stage (D17) to the harvesting stage (D38).

### 2.12. Statistical Analysis

All statistical analyses were performed using SPSS 27. A one-way analysis of variance (ANOVA) and Duncan’s test were used to determine differences in parameters measured in physical-chemical properties and cultivation tests (*p* < 0.05).

## 3. Results and Discussion

### 3.1. Synthesis and Characterization of WF and WF-Gel

**Figure 1:**
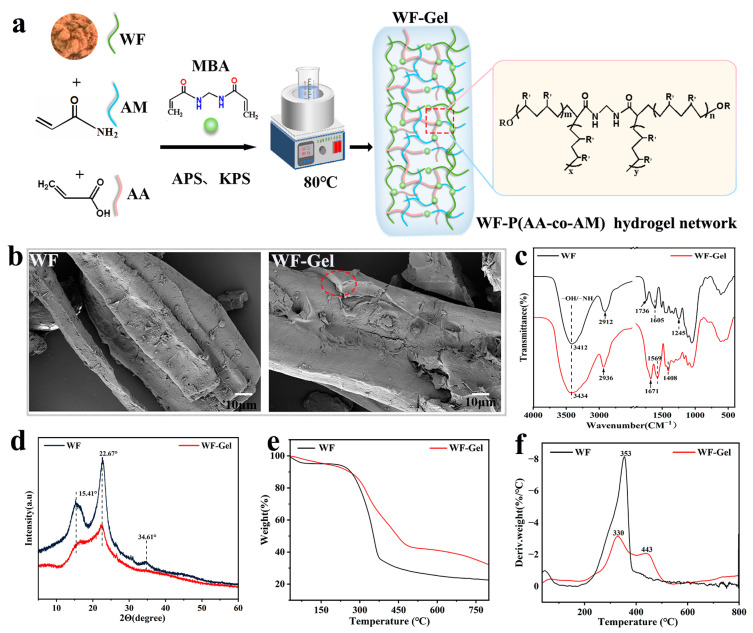
Overview of WF and WF-Gel characterization: (**a**) Hydrogel network formation, (**b**) SEM microstructure, (**c**) FTIR spectra, (**d**) XRD patterns, (**e**) TGA curves, and (**f**) DTG profiles of WF and WF-Gel.

Figure 1a presents the schematic illustration of the preparation of WF-Gel. The initiators (APS and KPS mixture) produce sulfate anion radicals under heating conditions, and these radicals abstract hydrogen atoms from the hydroxyl groups on the surface of WF, creating active sites where AA and AM are grafted to form and grow polymer chains. With the presence of MBA, a network structure is generated via a free-radical-inducing polymerization of grafted P(AA-co-AM).

Figure 1b shows SEM images at 1500× magnification. The WF exhibits a relatively smooth surface, whereas WF-Gel displays a thin, uniform coating on the fiber surface, corresponding to the grafted P(AA-co-AM) hydrogel layer.

The chemical structure of WF and WF-Gel was characterized by FT-IR, and their spectra are shown in Figure 1c. Compared with WF, WF-Gel exhibited characteristic shifts in several absorption bands, confirming successful grafting of P(AA-co-AM) onto the cellulose backbone. The O–H/N–H absorption band broadened and shifted slightly to 3434 cm^−1^, reflecting changes in the hydrogen-bonding environment after hydrogel incorporation. The C–H stretching vibration shifted from 2912 to 2936 cm^−1^, consistent with increased aliphatic content from the grafted copolymer [25]. The weak C=O band at 1736 cm^−1^ in WF (residual hemicellulose/ester groups) evolved into a strong peak at 1671 cm^−1^ in WF-Gel (amide I overlapping with carboxyl C=O), indicating the introduction of amide functionalities upon grafting [26]. The amide II band at 1569 cm^−1^ appeared, corresponding to N–H bending/C–N stretching of AM units, while the aromatic skeletal band at 1605 cm^−1^ decreased in intensity, indicating coverage by the polymer. The new 1408 cm^−1^ band corresponds to symmetric stretching of carboxylate (–COO^−^) from AA units [27]. Together, these spectral changes provide compelling evidence that P(AA-co-AM) chains were successfully grafted, resulting in the formation of the WF-Gel three-dimensional hydrogel network.

The chemical structure of WF and WF-Gel was characterized by FT-IR, and their spectrum are given in Figure 1c. Compared with WF, WF-Gel exhibited a broadened O–H/N–H absorption band that shifted slightly to 3434 cm^−1^, reflecting changes in the hydrogen-bonding environment after the introduction of the hydrogel network. The C–H stretching vibration also shifted from 2912 to 2936 cm^−1^, consistent with an increased content of aliphatic chains in the grafted copolymer [25]. Notably, the weak unconjugated C=O band at 1736 cm^−1^ in WF (attributed to residual hemicellulose or ester groups) evolved into a strong peak at 1671 cm^−1^ in WF-Gel, which is assigned to the amide I (C=O stretching) vibration overlapping with the carboxyl C=O group in P(AA-co-AM). This indicates the introduction of amide and carboxyl functionalities upon grafting [26]. In addition, a distinct peak at 1569 cm^−1^ was observed in WF-Gel, corresponding to the amide II vibration (N–H bending/C–N stretching) from AM units, while the aromatic skeletal band at 1605 cm^−1^ in WF decreased in intensity, suggesting coverage and interaction by the polymer layer. Furthermore, the new band at 1408 cm^−1^ in WF-Gel is attributed to the symmetric stretching of carboxylate (–COO^−^) groups from AA units, which was not evident in the WF control [27]. Taken together, the emergence and enhancement of the amide I (~1671 cm^−1^) and amide II (~1569 cm^−1^) bands, along with the appearance of the carboxylate signal at 1408 cm^−1^, provide compelling evidence that P(AA-co-AM) chains were successfully grafted onto the cellulose backbone, leading to the formation of the WF-Gel three-dimensional network.

Figure 1d shows the crystalline structure of WF and WF-Gel analyzed by XRD. For WF, three distinct diffraction peaks at 15.41°, 22.76°, and 34.61° were assigned to the (101), (002), and (040) crystal planes of the cellulose I lattice, respectively [28]. In contrast, WF-Gel showed a pronounced decrease in the intensities of the 15.41° and 22.76° peaks, accompanied by the disappearance of the 34.61° reflection. This loss of crystallinity suggests that the native polycrystalline cellulose structure was disrupted due to the introduction of the grafted P(AA-co-AM) network [29]. These XRD results further corroborate the FT-IR findings, confirming the successful grafting of P(AA-co-AM) chains onto the WF matrix.

The thermal stability of WF and WF-Gel was evaluated using thermogravimetric (TGA) and derivative thermogravimetric (DTG) analyses, as shown in Figure 1e,f. Both exhibited a multi-stage pyrolysis process. The initial stage of the thermogram in WF and WF-Gel ranging from 30 to 200 °C with a weight loss of 5.16% and 6.10%, respectively, was mainly attributed to the evaporation of water and unreacted reagents from the samples. The sharp decomposition rate of WF occurred in the temperature range of 200 °C to 400 °C and the maximum weight loss was recorded at 353 °C with around 50% weight loss, corresponding to degradation of WF components including hemicellulose, cellulose and lignin [30]. A reduced decomposition rate and extended main decomposition range (200–500 °C) were clearly observed in WF-Gel. The DTG curve showed a peak at 326 °C (~23% weight loss), mainly attributed to the degradation of wood components like hemicellulose and cellulose. Further degradation around 50% weight loss occurred at 443 °C owing to the exothermic chain scission of the grafted P(AA-co-AM) hydrogel [26,31]. The formation of a hydrogel network structure generated by MBA-crosslinked AM and AA chains onto the WF delayed the degradation process and enhanced the thermal stability of WF-Gel.

Overall, these structural and chemical characterizations collectively demonstrate that grafting P(AA-co-AM) onto the WF surface substantially alters its physicochemical properties. The introduction of the hydrogel network increases the density of hydrophilic groups (–COOH and –CONH_2_), as evidenced by the strengthened amide I, amide II, and carboxylate bands in the FT-IR spectra. Meanwhile, the reduced crystallinity observed in XRD confirms that the native cellulose domains were partially disrupted during grafting, which is consistent with the morphological changes revealed by SEM. Such structural modification provides additional sorption sites and forms a three-dimensional polymer matrix capable of binding and retaining water, thereby improving the hydrophilicity and wettability of the fiber surface. These observations are in line with earlier studies on hydrogel–cellulose composite systems [27], where polymer grafting was shown to enhance interfacial compatibility and promote more efficient interactions between cellulose fibers and hydrogel networks. The thermal analysis further corroborates these findings, indicating that the crosslinked hydrogel layer delays thermal degradation and improves the overall thermal stability of WF-Gel. In summary, these results confirm the successful construction of a grafted hydrogel layer on WF and illustrate that modification contributes to the enhanced functionality of the composite.

### 3.2. Characteristics of Peat Incorporated WF or WF-Gel as Growing Media

To evaluate the effect of incorporating WF or WF-Gel into peat on the basic characteristics of the growing substrate, results are presented in Table 1. The pH value and EC of peat were measured as 5.25 and 493 µs/cm, respectively. With the incorporation of WF or WF-Gel, both growing substrates Pt+5WF and Pt+5WF-Gel displayed a slight decrease in pH, probably owing to the acidic components in wood [8] and unreacted chemical reagents like AA (for WF-Gel). The EC value refers to the soluble salt concentration, and the significantly increased EC value in Pt+5WF-Gel is mainly attributed to unreacted reagents.

There were no significant differences in bulk density (BD) and total porosity (TP) of peat-based substrates amended with WF or WF-Gel compared to peat alone (Pt). As a fine particle size of WF (60 mesh) was used in this work, the mixed substrates exhibited slightly increased water-filled porosity and water-holding capacity compared with Pt [32]. This improvement is attributed to the three-dimensional crosslinked polymer network of the hydrogel, which efficiently absorbs and retains water [33,34].

### 3.3. Water Retention Capacity of Peat Substrate Mixed with WF or WF-Gel

**Figure 2:**
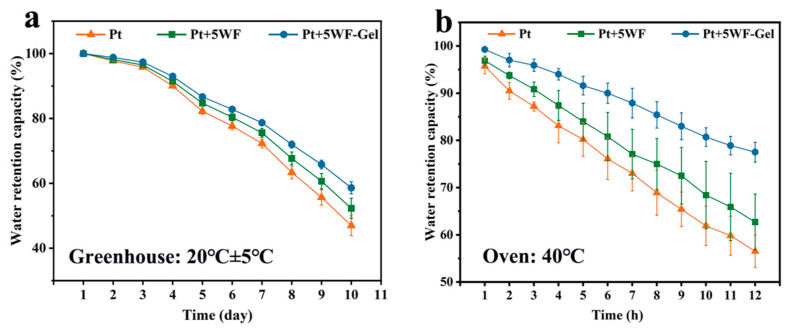
Water retention capacity of various growing substrates under different temperature conditions: (**a**) 20 ± 5 °C; (**b**) 40 °C.

It is crucial to evaluate the water retention behavior of WF-Gel for soilless substrate applications as the hydrogel-modified WF can guarantee that more water and nutrients are retained in the substrate, providing relatively long-term water release. Figure 2 shows the water retention capacity of Pt and Pt blended with WF or WF-Gel under different temperature conditions. All growing substrates gradually decrease in water content over time, regardless of whether they were under common or relatively high temperature conditions. Under the greenhouse testing conditions, the blended growing substrate Pt+5WF-Gel had the best water retention performance and retained nearly 60% water on the 10th day, which is 5% and 10% higher than Pt+5WF and Pt, respectively (Figure 2a). Especially under high temperatures (Figure 2b), Pt+5WF-Gel retained nearly 80% water at the 12th hour, while Pt and Pt+5WF retained around 55% and 60% at the same time, respectively. The presented results mean that the use of hydrogel-modified WF can significantly promote the long-term supply of water, reducing the watering frequency and increasing water-use efficiency [34]. This modification strategy of WF likely provides an efficient way to ameliorate the water retention behavior of plant fibrous material as a growing medium [16]. Furthermore, the superior water retention performance of WF-Gel can be attributed to the synergistic interaction between the porous structure of wood fiber and the high absorbency of the grafted hydrogel network. While WF alone improves rehydration due to its natural capillary channels, the hydrogel layer further traps water within the three-dimensional polymer matrix and slows evaporation. This dual mechanism has also been observed in other composite systems such as biochar–hydrogel and cellulose–hydrogel materials, which similarly demonstrate prolonged moisture release and reduced irrigation needs compared with untreated substrate components [20,21].

### 3.4. Chlorophyll Content and Relative Growth Rate

**Figure 3:**
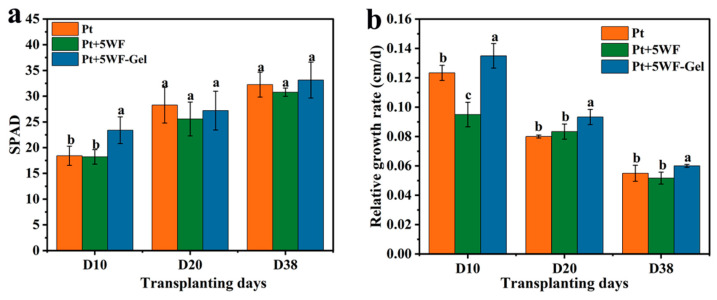
Chlorophyll content (**a**) in the leaves and relative growth rate (**b**) of choy sum in different substrate types after various transplanting days. Bars represent mean ± standard deviation (SD, *n* = 6). Different letters above the bars indicate significant differences (*p* < 0.05).

Figure 3 shows the chlorophyll content (a) and relative growth rate (b) of choy sum grown in different substrates, including peat (Pt), peat with WF (Pt+5WF), and peat with WF-Gel (Pt+5WF-Gel). Soilless substrates composed of peat and raw WF often limit plant growth under the same irrigation and fertilization regime due to nitrogen immobilization by WF [13,14,16,17,35]. As choy sum is highly sensitive to nitrogen availability, the temporary retention of soluble fertilizer by WF-Gel reduces nutrient leaching. It improves accessibility, thereby promoting faster canopy development and photosynthetic activity. The enhanced chlorophyll content and relative growth rate observed in WF-Gel substrates suggest that this composite provides a more favorable water–nutrient environment during the early growth stages. This observation is consistent with previous studies reporting that hydrogel incorporation increases nitrogen availability and enhances seedling establishment in leafy vegetables [36]. Choy sum, a C3 vegetable, is particularly dependent on nitrogen during early growth stages for the synthesis of proteins, nucleic acids, and chlorophyll, directly influencing biomass accumulation [37,38]. As shown in Figure 3a, plants grown in Pt+5WF-Gel exhibited significantly higher SPAD values than those in Pt and Pt+5WF at 10 days after transplanting (D10), indicating that the hydrogel network in WF-Gel effectively stores water and nutrients, particularly nitrogen, to support seedling growth. With the continued addition of soluble fertilizer, chlorophyll content differences among substrates became negligible due to sufficient nitrogen supply.

A similar trend was observed for relative growth rate (RGR) in Figure 3b. Choy sum in the WF-Gel substrate showed the highest RGR (0.135 cm/d) at D10, significantly exceeding that in Pt (control) and Pt+5WF, whereas the substrate containing raw WF caused growth inhibition (RGR = 0.095 cm/d). As the experiment progressed, differences in RGR among substrates gradually decreased; however, plants in Pt+5WF-Gel continued to exhibit significantly higher growth rates at D20 and D38. These results demonstrate that the hydrogel modification of WF enhances plant growth by improving water and nutrient retention, in agreement with previous reports using hydrogel-amended substrates [22,34,39].

### 3.5. Plant Growth Index and Nutrient Content at Harvest

**Figure 4:**
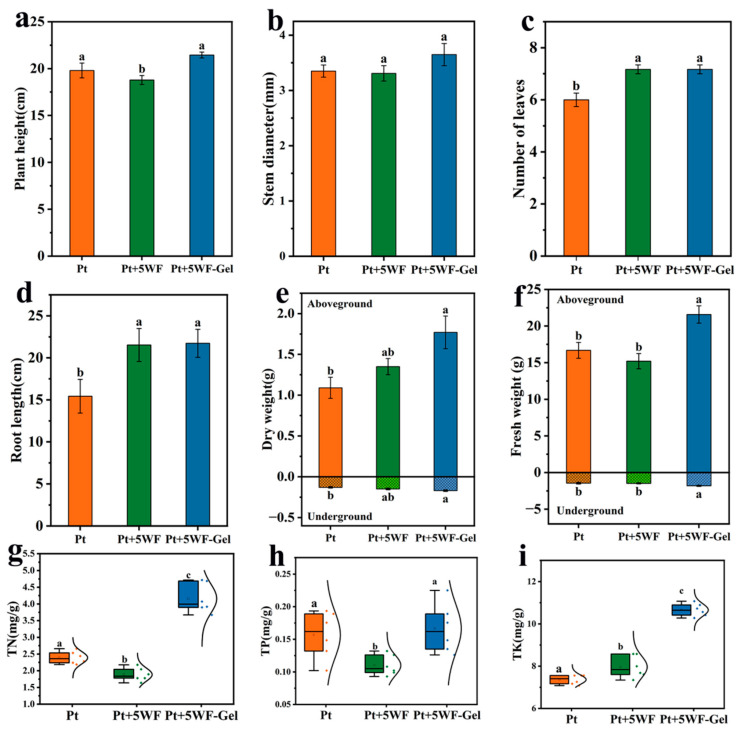
The growth indices of choy sum include (**a**) plant height, (**b**) stem diameter, (**c**) number of leaves, (**d**) root length, (**e**) fresh weight, (**f**) dry weight, (**g**) total nitrogen content (TN), (**h**) total phosphorus content (TP), and (**i**) total potassium content (TK). Bars represent mean ± standard deviation (SD, *n* = 6). Different letters above the bars indicate significant differences (*p* < 0.05).

To evaluate the choy sum cultivated in different growing media, a series of growth indices were measured on the 38th day, including morphological parameters (plant height, stem diameter, number of leaves and root length), biomass accumulation (shoot and root fresh/dry weight), and plant nutrients (total N/P/K content), and the results are shown in Figure 4. As discussed in the previous section, the SPAD content and relative growth rate of choy sum in various growing media except Pt+5WF-Gel showed no significant differences with the continuing supply of soluble fertilizer. After 38 transplanting days, choy sum exhibited a similar growth status, as shown in the digital image (Figure S2).

Growth performance of choy sum cultivated in different substrates is presented as follows: Figure 4a—plant height, Figure 4b—stem diameter, Figure 4c—number of leaves, and Figure 4d—root length. Choy sum grown in Pt+5WF-Gel exhibited significantly greater plant height compared to the control (Pt) and Pt+5WF. Although stem diameter, number of leaves, and root length showed no significant differences between Pt+5WF and Pt+5WF-Gel, both treatments outperformed the control, with Pt+5WF-Gel showing slightly better leaf number and root length. These improvements may be attributed to the enhanced water and nutrient transport provided by WF [40,41,42], while the incorporation of WF-Gel further supplied sufficient storage capacity for water and nutrients around the root zone [22], which is also reflected in the WHC and water retention results (Table 1). Another possible explanation is that WF and WF-Gel can provide more macropores for root respiration due to the fluffy nature of WF and the swollen nature of the hydrogel during cultivation.

Significant differences in biomass accumulation of harvested choy sum grown in Pt+5WF-Gel compared to control Pt and Pt+5WF can be observed in Figure 4e,f. Both the fresh and dry weights of the aboveground parts of choy sum in Pt+5WF-Gel were around 40% and 30% higher than those in Pt+5WF, respectively, while the fresh and dry weights of the underground parts of choy sum in Pt+5WF-Gel were 22% and 13% higher than those in Pt+5WF, respectively. With the combination of nutrient content in Figure 4g–i, choy sum planted in Pt+5WF-Gel displayed significantly higher TN, TP, and TK, especially TN and TK. As demonstrated by Yuichi Mori, the application of hydrogel as a growing medium generally creates water stress around the root environment, which can promote the generation of fine roots and synthesize more sugar and amino acids to raise the intracellular osmotic pressure for transferring water and nutrients from the hydrogel to the plant by an osmotic gradient [43]. Therefore, the hydrogel modification strategy of WF can promote plant growth and nutrient-use efficiency by creating a slightly water-stressed environment.

### 3.6. Irrigation Water-Use Efficiency

**Figure 5:**
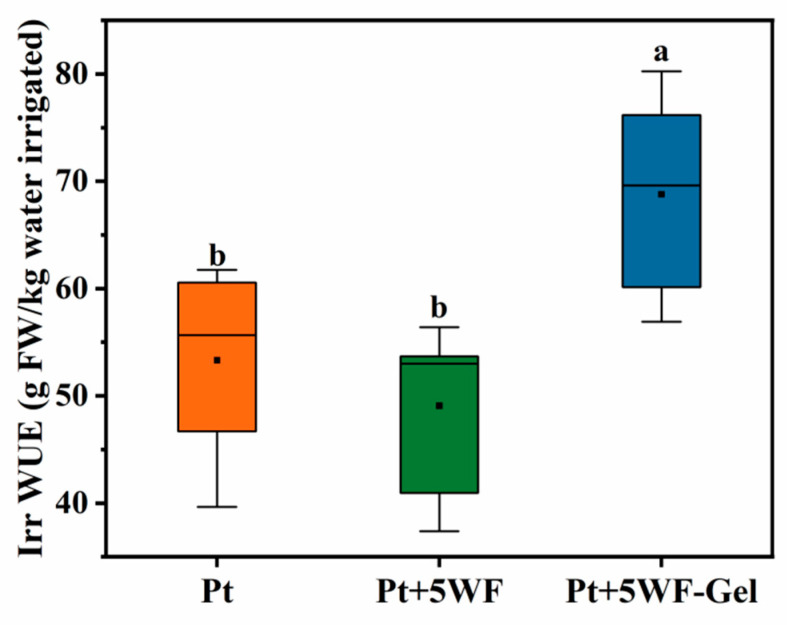
Irrigation water-use efficiency (Irr WUE) of choy sum under different treatments. The black squares indicate the mean values. Bars represent mean ± standard deviation (SD, *n* = 6). Different letters above the bars indicate significant differences (*p* < 0.05).

To address whether the superior growth performance of choy sum planted in WF-Gel amendment could be attributed to irrigation water-use efficiency, Irr WUE was calculated and presented in Figure 5. The choy sum in Pt+5WF-Gel was watered 5 times (a total of 340 mL soluble nutrient solution) prior to harvest and produced 23.39 ± 3.1 g shoot weight per kg of water irrigated, compared to choy sum in Pt, which showed a significant decrease to 16.69 ± 2.67 g, and Pt+5WF, which decreased to 18.13 ± 2.93 g with the same volume of soluble nutrient solution. These results indicate that WF-Gel strongly promotes the water and nutrient efficiency of peat-based substrates, in agreement with similar studies [22].

## 4. Conclusions

In this study, a wood fiber–hydrogel composite (WF-Gel) was synthesized by grafting P(AA-co-AM) onto raw wood fibers in a single-step procedure to enhance the water- and nutrient-use efficiency of wood fiber in soilless cultivation. Successful fabrication of WF-Gel was confirmed via SEM, FTIR, XRD, and TGA analyses. When incorporated at 5% into peat-based substrates, WF-Gel substantially improved water-filled porosity (67.14%), water-holding capacity (519.60%), and overall water retention relative to peat alone or peat amended with unmodified WF. Pot experiments further demonstrated that substrates containing WF-Gel significantly enhanced SPAD values and relative growth rates of choy sum during early growth stages. Additionally, biomass accumulation and total N, P, and K contents in choy sum were markedly higher in WF-Gel-amended substrates compared with peat or peat plus WF. The irrigation water-use efficiency was also significantly improved, highlighting the potential advantage of WF-Gel in optimizing water and nutrient utilization in soilless cultivation systems. In our future research, the development of WF-Gel or related composites will be explored as a stand-alone eco-friendly alternative to peat for sustainable soilless substrates.

## Figures and Tables

**Table 1:** Basic properties of peat substrate mixed with WF or WF-Gel as growing media.

Growing Substrate Type	pH	EC µs/cm	BD g/cm^3^	TP %	AFP %	WFP %	WHC %
Pt	5.25 ±0.049 ^a^	493 ±4 ^b^	0.16 ±0.00 ^a^	98.64 ±1.65 ^a^	57.28 ±7.63 ^a^	41.36 ±8.91 ^b^	358.51 ±55.66 ^b^
Pt+5WF	5.11 ±0.025 ^b^	462 ±5 ^b^	0.16 ±0.00 ^a^	97.02 ±4.98 ^a^	52.36 ±3.04 ^a^	44.66 ±3.16 ^b^	379.15 ±19.73 ^b^
Pt+5WF-Gel	5.08 ±0.053 ^b^	928 ±11 ^a^	0.16 ±0.00 ^a^	99.15 ±10.73 ^a^	32.01 ±11.52 ^b^	67.14 ±0.83 ^a^	519.60 ±5.20 ^a^

Note: EC: electrical conductivity, BD: dry bulk density, TP: total porosity, AFP: air-filled porosity, WFP: water-filled porosity; WHC: water-holding capacity. *p* < 0.05; *n* = 3. Values following the same letter mean no significant difference.

## Data Availability

The original contributions presented in this study are included in the article/Supplementary Material. Further inquiries can be directed to the corresponding authors.

## Supplementary Materials

The following supporting information can be downloaded at https://www.mdpi.com/article/10.3390/ma18235461/s1, Figure S1: Effect of synthesis parameters on the water and saline absorbency of the resin; Figure S2: Photographs of choy sum growth; Table S1: The orthogonal experiment for modification of WF; Table S2: The orthogonal experiment results; Table S3: Analysis of orthogonal block test results.

## References

[1] Gruda N. (2019). Increasing Sustainability of Growing Media Constituents and Stand-Alone Substrates in Soilless Culture Systems. Agronomy.

[2] Savvas D., Gruda N. (2018). Application of Soilless Culture Technologies in the Modern Greenhouse Industry–A Review. Eur. J. Hortic. Sci..

[3] Dittrich C., Pecenka R., Løes A.-K., Cáceres R., Conroy J., Rayns F., Schmutz U., Kir A., Kruggel-Emden H. (2021). Extrusion of Different Plants into Fibre for Peat Replacement in Growing Media: Adjustment of Parameters to Achieve Satisfactory Physical Fibre-Properties. Agronomy.

[4] Gaudig G. (2020). Sphagnum Growth and Its Perspectives for Sphagnum Farming. Ph.D. Thesis.

[5] Venkataramani S., Kafle A., Singh M., Singh S., Simpson C., Siebecker M.G. (2023). Greenhouse Cultivation of Cucumber (*Cucumis sativus* L.) in Standard Soilless Media Amended with Biochar and Compost. HortScience.

[6] Zabaleta R., Sánchez E., Fabani P., Mazza G., Rodriguez R. (2024). Almond Shell Biochar: Characterization and Application in Soilless Cultivation of Eruca Sativa. Biomass Convers. Biorefin..

[7] Jackson B.E. (2018). Challenges and Considerations of Using Wood Substrates: Physical Properties. Greenh. Grow..

[8] Jackson B.E., Bartley P. (2017). Wood Substrates: The Plant’s Perspective. Grow. Talks.

[9] Poleatewich A., Michaud I., Jackson B., Krause M., DeGenring L. (2022). The Effect of Peat Moss Amended with Three Engineered Wood Substrate Components on Suppression of Damping-Off Caused by Rhizoctonia Solani. Agriculture.

[10] Lahti S. (2022). The Effects of Added Wood Fibre in Peat-Based Growing Medium on Petunia x Hybrida. Master’s Thesis.

[11] Woznicki T., Kusnierek K., Roos U.M., Andersen S., Zimmer K., Sønsteby A. Exploration of Alternative Growing Media in Strawberry Production with Focus on Wood Fiber from Norway Spruce. Proceedings of the III International Symposium on Growing Media, Composting and Substrate Analysis.

[12] Kusnierek K., Heltoft P., Møllerhagen P.J., Woznicki T. (2023). Hydroponic Potato Production in Wood Fiber for Food Security. npj Sci. Food.

[13] Čepulienė R., Butkevičienė L.M., Skinulienė L., Steponavičienė V. (2022). Response of Cucumbers (*Cucumis sativus* L.) to Waste Wood Fiber Substrates and Additional Nitrogen Fertilization. Plants.

[14] Harris C. (2019). Evaluating Wood Fiber Soilless Substrates for Effects on Plant Performance and Nutrient Management in Container Crops. Master’s Thesis.

[15] Komosa A., Piróg J., Kleiber T. (2009). Changes of Macro and Micronutrients Contents in the Root Environment of Greenhouse Tomato Grown in Fiber Wood. J. Fruit Ornam. Plant Res..

[16] Woznicki T., Jackson B.E., Sønsteby A., Kusnierek K. (2023). Wood Fiber from Norway Spruce—A Stand-Alone Growing Medium for Hydroponic Strawberry Production. Horticulturae.

[17] Harris C.N., Dickson R.W., Fisher P.R., Jackson B.E., Poleatewich A.M. (2020). Evaluating Peat Substrates Amended with Pine Wood Fiber for Nitrogen Immobilization and Effects on Plant Performance with Container-Grown Petunia. HortTechnology.

[18] Ma L., Chai C., Wu W., Qi P., Liu X., Hao J. (2023). Hydrogels as the Plant Culture Substrates: A Review. Carbohydr. Polym..

[19] Ramli R.A., Lian Y.M., Nor N.M., Azman N.I.Z. (2019). Synthesis, Characterization, and Morphology Study of Coco Peat-Grafted-Poly(Acrylic Acid)/NPK Slow Release Fertilizer Hydrogel. J. Polym. Res..

[20] Wu Y., Brickler C., Li S., Chen G. (2021). Synthesis of Microwave-Mediated Biochar-Hydrogel Composites for Enhanced Water Absorbency and Nitrogen Release. Polym. Test..

[21] Cao L., Li N. (2021). Activated-Carbon-Filled Agarose Hydrogel as a Natural Medium for Seed Germination and Seedling Growth. Int. J. Biol. Macromol..

[22] Tan W.K., Zhu J., Lim J.Y., Gao Z., Loh C.S., Li J., Ong C.N. (2021). Use of Okara-Derived Hydrogel for Enhancing Growth of Plants by Minimizing Leaching and Locking Nutrients and Water in Growing Substrate. Ecol. Eng..

[23] Gessert G. (1976). Measuring a Medium’s Air Space and Water Holding Capacity. Ornam. Northwest.

[24] Zhang D., Peng Q., Yang R., Lin W., Wang H., Zhou W., Qi Z., Ouyang L. (2023). Slight Carbonization as a New Approach to Obtain Peat Alternative. Ind. Crop. Prod..

[25] Shah L.A., Khan M., Javed R., Sayed M., Khan M.S., Khan A., Ullah M. (2018). Superabsorbent Polymer Hydrogels with Good Thermal and Mechanical Properties for Removal of Selected Heavy Metal Ions. J. Clean. Prod..

[26] Zhao B., Jiang H., Lin Z., Xu S., Xie J., Zhang A. (2019). Preparation of Acrylamide/Acrylic Acid Cellulose Hydrogels for the Adsorption of Heavy Metal Ions. Carbohydr. Polym..

[27] Zheng M., Lian F., Zhu Y., Zhang Y., Liu B., Zhang L., Zheng B. (2019). pH-Responsive Poly (Xanthan Gum-g-Acrylamide-g-Acrylic Acid) Hydrogel: Preparation, Characterization, and Application. Carbohydr. Polym..

[28] Yi C., Niu H.-Y., Sui L., Zhu J.-J., Tian Y., Niu C.-G., Chen Z.-L., Wei H., Huang D.-W. (2025). A Low-Cost Bio-Based Cellulose Composite Hydrogel with Cross-Linked Structures for Efficient Capture of Heavy Metal Ions. Sep. Purif. Technol..

[29] Mohd Amin M.C.I., Ahmad N., Pandey M., Jue Xin C. (2014). Stimuli-Responsive Bacterial Cellulose-g-Poly(Acrylic Acid-Co-Acrylamide) Hydrogels for Oral Controlled Release Drug Delivery. Drug Dev. Ind. Pharm..

[30] Carrillo I., Mendonça R.T., Ago M., Rojas O.J. (2018). Comparative Study of Cellulosic Components Isolated from Different Eucalyptus Species. Cellulose.

[31] Zaharia A., Radu A.-L., Iancu S., Florea A.-M., Sandu T., Minca I., Fruth-Oprisan V., Teodorescu M., Sarbu A., Iordache T.-V. (2018). Bacterial Cellulose-Poly(Acrylic Acid- Co-N, N ′-Methylene-Bis-Acrylamide) Interpenetrated Networks for the Controlled Release of Fertilizers. RSC Adv..

[32] Mizell A. (2024). Evaluation of Wood Fibers Derived from Different Tree Species and Processing Methods on Crop Growth and Microbial Activity in Soilless Substrates. Master’s Thesis.

[33] Das D., Bhattacharjee S., Bhaladhare S. (2023). Preparation of Cellulose Hydrogels and Hydrogel Nanocomposites Reinforced by Crystalline Cellulose Nanofibers (CNFs) as a Water Reservoir for Agriculture Use. ACS Appl. Polym. Mater..

[34] Qin C., Kan X., Xu D., Zhao Y., Qi Y., Wu N., Xu W. (2025). Adjustable P(AM-Co-NIPAM)/Gelatin Hydrogel Soilless Cultivation Substrates for Soybean Seedling and Root Growth. Ind. Crop. Prod..

[35] Vandecasteele B., Muylle H., De Windt I., Van Acker J., Ameloot N., Moreaux K., Coucke P., Debode J. (2018). Plant Fibers for Renewable Growing Media: Potential of Defibration, Acidification or Inoculation with Biocontrol Fungi to Reduce the N Drawdown and Plant Pathogens. J. Clean. Prod..

[36] Katiane S.S.B., Cleiton G.S.B., Guilherme G.D.S., Edilson C. (2015). Effects of Hydrogel and Nitrogen Fertilization on the Production of Arugula in Successive Crops. Afr. J. Agric. Res..

[37] Greenwood D.J., Lemaire G., Gosse G., Cruz P., Draycott A., Neeteson J.J. (1990). Decline in Percentage N of C3 and C4 Crops with Increasing Plant Mass. Ann. Bot..

[38] Valenzuela H. (2024). Optimizing the Nitrogen Use Efficiency in Vegetable Crops. Nitrogen.

[39] Zhang Z., Zhu J., Song X., Wen Y., Zhu C., Li J. (2023). Biomass-Based Single- and Double-Network Hydrogels Derived from Cellulose Microfiber and Chitosan for Potential Application as Plant Growing Substrate. Carbohydr. Polym..

[40] Schulker B.A., Jackson B.E. Impact of Wood Fiber Substrate Additions on Water Capture through Surface and Subsurface Irrigation. Proceedings of the XXXI International Horticultural Congress (IHC2022): International Symposium on Innovative Technologies and Production.

[41] Michel J.-C., Durand S., Jackson B.E., Fonteno W.C. Analyzing Rehydration Efficiency of Hydrophilic (Wood Fiber) vs. Potentially Hydrophobic (Peat) Substrates Using Different Irrigation Methods. Proceedings of the II International Symposium on Growing Media, Soilless Cultivation, and Compost Utilization in Horticulture.

[42] Durand S., Jackson B.E., Fonteno W.C., Michel J.-C. (2021). The Use of Wood Fiber for Reducing Risks of Hydrophobicity in Peat-Based Substrates. Agronomy.

[43] Mori Y. (2013). New Agro-Technology (Imec) by Hydrogel Membrane. React. Funct. Polym..

