# Supplementary material for: Facile Fabrication of Wood Fiber–Hydrogel Composites for Enhanced Water and Nutrient Efficiency in Soilless Cultivation
Source: Materials (Basel). 2025 Dec 4;18(23):5461. doi: 10.3390/ma18235461 (PMC12693702; doi:10.3390/ma18235461)
Supplement: Supplementary file 1 [file materials-18-05461-s001.zip › materials-3970221-supplementary.pdf]

Facile Fabrication of Wood Fiber–Hydrogel Composites for Enhanced Water and Nutrient Efficiency in Soilless Cultivation

Zhengyong Yang [2], Yao Qu [1], Longqing Chen [1], Huishu Mo [3], Chunyu Ji [3], Nicolas Brosse [4], Mahdi Mubarok [5], Xiaojian Zhou [2,*], Yining Di [3,*] and Jingjing Liao [1,*]

1 Key Laboratory of Vegetable Biology of Yunnan Province, College of Landscape and Horticulture, Yunnan Agricultural University, 650201 Kunming, Yunnan, China

2 College of Materials and Chemical Engineering, Southwest Forestry University, 650224 Kunming, Yunnan, China

3 College of Resources and Environment, Yunnan Agricultural University, 650201 Kunming, Yunnan, China

4 Laboratoire d'Etudes et de Recherche sur le Matériau Bois (LERMAB), Faculté des Sciences et Technologies, l'Université de Lorraine, 54000 Vandœuvre-lès-Nancy, France

5 Faculty of Forestry and Environment, Department of Forest Products, IPB University, Bogor 16680, Indonesia

Preliminary orthogonal experiments

For confirming the optimum parameters for wood fiber modification, an orthogonal experiment of L9(3^4) was designed as follow:

Table S1 the orthogonal experiment for modification of WF

Factor	AA Neutrality/%	AAm/g	MBA/g	Initiator/g
1	60%	8	1	0.2
2	70%	12	2	0.5
3	80%	16	3	0.8

Note: Acrylic acid (AA), acrylamide (AM), and N,N'-methylenebisacrylamide (MBA), initiator is the mixture of APS:KPS in a weight ratio of 1:1

Table S2 The orthogonal experiment results

**Experiment No. / Factor**	A（AA-Neutrality）	B(AM/g)	C(MBA/g)	D(Initiator/g)	Water absorbency (g/g)	Saline absorbency (g/g)
1	1	1	1	1	59.58	11.9
2	1	2	2	2	73.43	21.47
3	1	3	3	3	47.43	19.13
4	2	1	2	3	59.28	15.45
5*	2	2	3	1	101.13	22.05
6	2	3	1	2	50.08	19.18
7	3	1	3	2	52.43	19.3
8	3	2	1	3	87.88	23.23
9	3	3	2	1	81.2	23.7

Table S3 Analysis of orthogonal block test results

Items	Water absorbency(g/g)				Saline absorbency(g/g)			
	A	B	C	D	A	B	C	D
k1	60.15	57.1	65.85	80.64	17.5	15.55	18.1	19.22
k2	70.16	87.48	71.3	58.65	18.89	22.25	20.21	19.98
k3	73.84	59.57	67	64.86	22.08	20.67	20.16	19.27
Range	13.69	30.38	5.15	31.99	4.58	6.7	2.11	0.76
Optimum combination	B2C2A3D2				A3B2C2D2			

Tables S2 and **S3** summarize the L9(3^4) orthogonal experimental results for optimizing the effects of various factors on the water and saline absorbency of the wood fiber-based superabsorbent resin. As shown in **Table S2**, the maximum water absorbency of 101.13 g/g in distilled water was achieved under the experimental

conditions of 70% AA neutralization degree, 12 g of AAm, 3 g of crosslinker, and 0.2 g of initiator. For saline absorbency, the optimal experimental condition was found in **run #9**, yielding an absorbency of 23.7 g/g in 0.9% NaCl solution.

Table S3 further reveals the relative influence of different factors. Among them, the initiator dosage had the greatest effect on the water absorbency of the resin, whereas the crosslinker dosage had the least. The optimal formulation for water absorbency was identified as B2C2A3D2. For saline absorbency in 0.9% NaCl solution, the optimal condition was A3B2C2D2, corresponding to 80% AA neutralization degree, 12 g of AAm, 2 g of crosslinker, and 0.5 g of initiator.

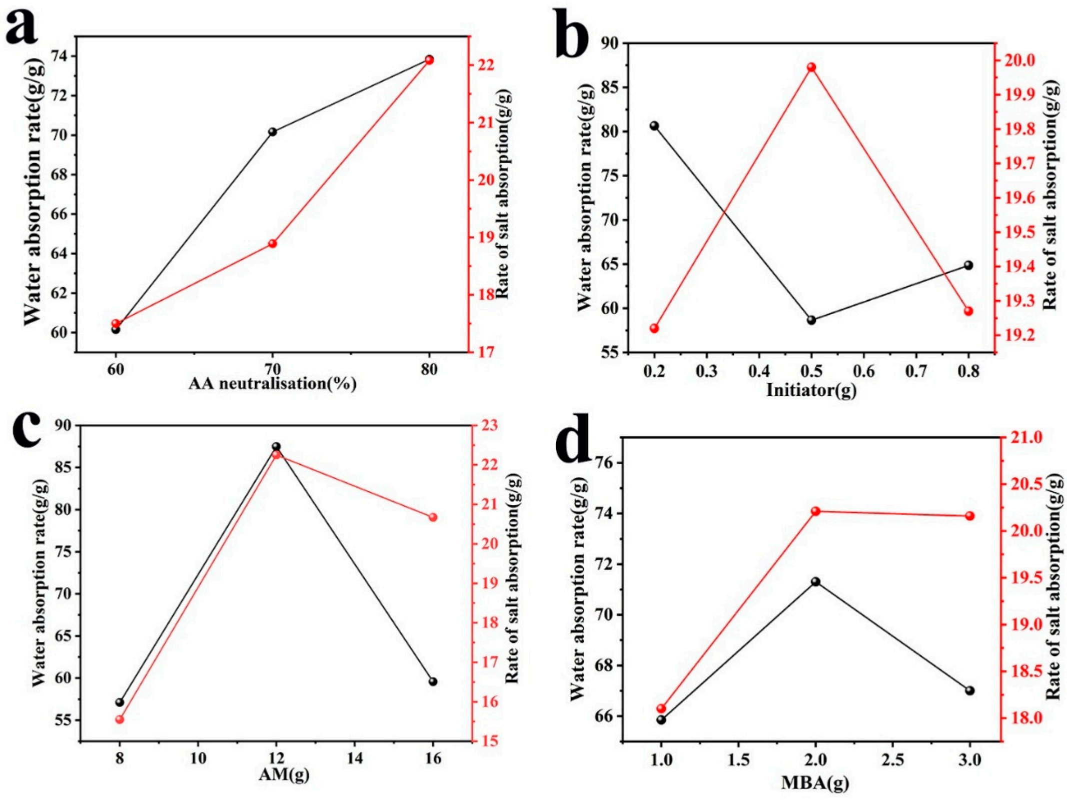

Figure S1. Effect of synthesis parameters on the water and saline absorbency of the resin: (a) AA neutralization degree; (b) initiator dosage; (c) AM dosage; (d) initiator dosage.

Figure S1 illustrates the variation trends of water absorbency of the resin with different influencing factors. As shown in **Figure S1(a)**, both the water absorbency in distilled water and the swelling capacity in 0.9% NaCl solution increased with the

degree of neutralization of acrylic acid. This trend is consistent with the general behavior observed in acrylic acid-based superabsorbent resins. The underlying mechanism can be attributed to the fact that cellulose tends to undergo hydrolysis under strongly acidic conditions, producing glucose molecules, while acrylic acid itself is prone to self-polymerization under acidic conditions. Both of these reactions are unfavorable for the grafting copolymerization process. Therefore, an appropriate increase in the neutralization degree of acrylic acid not only enhances the ionic charge density inside the resin but also raises the osmotic pressure and strengthens the electrostatic repulsion between carboxylate anions. These effects collectively contribute to the improved water and saline absorbency of the resin.

Figure S1(b) illustrates the effect of initiator dosage on the swelling capacity of the resin in both distilled water and 0.9% NaCl solution. The results showed that the water absorbency first decreased and then increased with increasing initiator concentration, whereas the saline absorbency initially increased and then declined. This phenomenon can be explained by the fact that the amount of initiator directly affects the molecular weight of the polymer, the crosslinking density, and the polymerization rate. At low initiator concentrations, only a limited number of free radicals are generated on the wood fiber backbone, resulting in insufficient grafting. Consequently, many monomers are merely physically adsorbed onto the resin surface and can be easily leached out during swelling, which significantly reduces the water absorbency of the resin. Conversely, when the initiator concentration is excessively high, the crosslinking density becomes too great, thereby restricting the network expansion and impairing both water and saline absorbency.

Figure S1(c) shows the effect of acrylamide (AM) dosage on the water and saline absorbency of the resin. Clearly, both water absorbency in distilled water and saline absorbency in 0.9% NaCl initially increased and then decreased with increasing AM content. This behavior can be attributed to the synergistic interaction between the nonionic hydrophilic group (–$CONH_2$) and the anionic water-absorbing group (–COONa) during resin synthesis, which mitigates the co-ion and salt effects and

enhances water and saline absorbency. However, as the AM content continues to increase, the absorbency gradually declines because the water-absorbing ability of –$CONH_2$ is much lower than that of –COOH. Excessive AM increases the proportion of –$CONH_2$ while reducing –COOH, ultimately leading to a decrease in water and saline absorbency. These results indicate that the amount of the second grafting monomer AM can improve the resin's water and saline absorbency, but higher dosages are not necessarily better. The optimal AM content should be adjusted in coordination with the AA dosage. In this study, the maximum water and saline absorbency of 87.48 g/g was achieved at an AM dosage of 12 g.

Figure S1(d) illustrates the effect of crosslinker (MBA) dosage on the water and saline absorbency of the resin. As shown, the water absorbency in distilled water initially increased and then decreased with increasing MBA content, while the saline absorbency in 0.9% NaCl exhibited a more gradual decline. The water absorbency of the resin is primarily determined by the number of water-absorbing groups and the crosslinking density. By adjusting the MBA dosage, the molecular chain length between crosslinking points can be controlled, thereby modulating the size of the resin network and the overall crosslinking density. At low MBA concentrations, insufficient micro-crosslinking occurs, making it difficult to form a stable polymer network. Uncrosslinked molecules may dissolve further in water, leading to reduced water and saline absorbency. Conversely, excessive MBA results in an overly dense network with high crosslinking density, increased gel stiffness, and restricted swelling, which also diminishes water and saline absorbency. In this study, the optimal MBA dosage was determined to be 2 g.

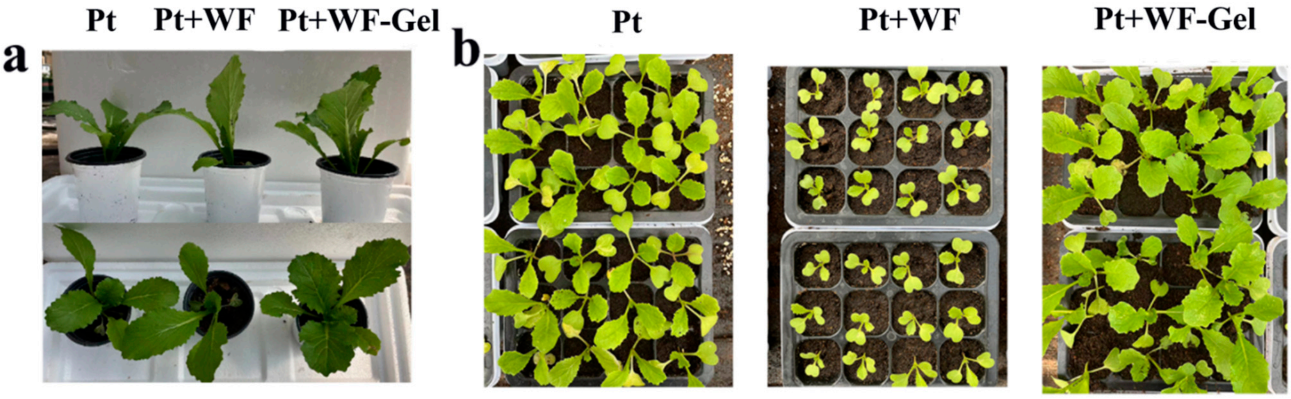

Figure S2. Photographs of choy sum growth: (a) plants after 38 days of transplantation; (b) seedlings after 16 days of cultivation.
